# 44 Sexual Function, Body Image, and Quality of Life in Genital Burns

**DOI:** 10.1093/jbcr/iraf019.044

**Published:** 2025-04-01

**Authors:** Dania Johnson, Andrew Humbert, Kimberly Roaten, Shelley Wiechman, Jeffrey Schneider, Haig Yenikomshian

**Affiliations:** University of California Keck School of Medicine; University of Washington; University of Texas Southwestern; Harborview Medical Center, University of Washington; Spaulding Rehabilitation Hospital, Harvard Medical School; University of California Keck School of Medicine

## Abstract

**Introduction:**

Burn injuries can impact both functional and psychosocial quality of life. Genital burns may uniquely impact anatomy and sensation crucial for sexual experience. Despite this potential impact, research remains limited. This study aims to explore the impact of genital burns on patient-reported outcomes body image, sexual satisfaction, and health-related quality of life, tracking these outcomes measured at 6,12, and 24 months post-discharge.

**Methods:**

This is a retrospective analysis of adult participants over the age of 18 from 2016 to 2024 in a multicenter longitudinal patient-reported outcome database. Linear mixed-effect models assessed the impact of genital burns on PROMIS-29 outcomes (anxiety, depression, fatigue, pain, function, sleep, sexual satisfaction) and BSHS body image at 12 and 24 months, adjusting for time, sex, age, race, ethnicity, and TBSA. Additional models were fit interacting time and presence of genital burn to understand how recovery trajectories may differ.

**Results:**

4,594 participants met inclusion criteria with 649 having genital burns and 3,945 without. After adjustment, there was no significant difference in levels of anxiety, depression, fatigue, pain interference, physical function, sexual satisfaction, sleep disturbance, or body image.

After adding the interaction term, significant differences in change scores from 6 to 12 months were observed for sleep disturbance (p = 0.042) and body image scores (p = 0.026). Those with genital burns had a smaller reduction in sleep disturbance (-0.09) compared to those without perineal burns, who had a greater reduction (-1.79). For body image, individuals with genital burns experienced a slight decrease in scores (0.2), while those without showed a slight increase (0.41).

At 24 months, significant differences in the trajectory for sleep disturbance were not maintained, however differences in body image scores were still significant (p = 0.001). Significant differences in the trajectory for depression at 24 months were also present (p = 0.036), with those with genital burns experiencing a 1.22-point increase, whereas those without showed a 0.60-point reduction from 6 months.

No significant differences in trajectories were found for anxiety, fatigue, pain interference, physical function, or sexual satisfaction.

**Conclusions:**

Genital burns significantly affect recovery, particularly in mental health and body image. While burn size and age matter, recovery in depression and body image is slower compared to non-genital burns, even two years post-injury. However, physical functioning and sexual satisfaction recover similarly in both groups, highlighting the need for specialized post-burn sexual health training.

**Applicability of Research to Practice:**

This study highlights the importance of sexual function and body image screening in burn recovery to support the psychosocial well-being of burn survivors.

**Funding for the Study:**

The contents of this abstract were developed under a grant from the National Institute on Disability, Independent Living, and Rehabilitation Research (NIDILRR grant number 90DPBU0007). NIDILRR is a Center within the Administration for Community Living (ACL), Department of health and Human Services (HHS). The contents of this abstract do not necessarily represent the policy of NIDILRR, ACL, or HHS, and you should not assume endorsement by the Federal Government.

